# A small 3D-printing model of macroadenomas for endoscopic endonasal surgery

**DOI:** 10.1007/s11102-018-0927-x

**Published:** 2018-11-30

**Authors:** Xing Huang, Zhen Liu, Xuan Wang, Xu-dong Li, Kai Cheng, Yan Zhou, Xiao-bing Jiang

**Affiliations:** Department of Neurosurgery, Union Hospital, Tongji Medical College, Huazhong University of Science and Technology, Wuhan, 430022 Hubei China

**Keywords:** 3D printing, Endoscopic endonasal approach, Macroadenomas, Anatomical modeling, Surgical simulation

## Abstract

**Purpose:**

This paper examines the application of 3D printing technology in the endoscopic endonasal approach for the treatment of macroadenomas.

**Methods:**

We have retrospectively analysed 20 patients who diagnosed with macroadenoma underwent endoscopic transsphenoidal surgery in Wuhan Union hospital from January 2017 to May 2017. Among the 20 patients, 10 patients received the service of 3D printing technology preoperatively. The data of 3D processing and clinical result were recorded for further evaluation.

**Results:**

The 10 patients who received the service had a successful 3D printed model of their tumors, it shows the anatomy of sphenoid sinus, tumor location which were in good agreement with our intraoperative observations. The 10 patients who received the service had a less operation time (127.0 ± 15.53 vs. 143.40 ± 17.89), blood loss (159.90 ± 12.31 vs. 170.00 ± 29.06) and less postoperative complication rate (20% vs. 40%). the design time of the 3D images varies 2 h 10 min to 4 h 32 min. the printing time of the 3D models varies 10 h 12 min to 22 h 34 min.

**Conclusions:**

The use of 3D printing technology has unquestionable potential applications to endoscopic endonasal approach for macroadenomas, in particular reflecting the complicated anatomy of sphenoid sinus and tumor location. Owing to the advantages of 3D printing technology, it may help the patients get a good prognosis.

## Introduction

The endoscopic endonasal transsphenoidal approach is a common procedure used for skull middle line diseases, such as pituitary adenomas (PAs) and chordoma [1–6]. Its advantages are a rapid recovery, shorter operation time [7], less blood loss, better visual results, less trauma to the brain, and fewer seizures [8]. However, this approach is still a challenging procedure for most neurosurgeons [9], especially for beginners. Potential disadvantages of this procedure include the relatively restricted working space and the danger of an inadequate dural repair with cerebrospinal fluid (CSF) leakage and potential for meningitis [10]. Several factors influence the outcome of endoscopic endonasal surgery, including the tumor volume, patient age, lesion location, and sphenoid pneumatolysis [11]. An accurate model of the target tumor structure is a major prerequisite for a successful PAs resection, expecially for macroadenomas, as this may avoid disastrous complications due to suboptimal treatment.

MRI and CT are the benchmark imaging modalities to delineate cerebral PAs in the planning of endoscopic endonasal surgeries. These techniques can provide information such as tumor position and blood supply [12, 13]. The radiographic evaluation of the skull base anatomy and its relationship to associated tumors is critical for both preoperative planning and intraoperative guidance. Thus, CT and MRI play significant roles in guiding endoscopic endonasal procedures. Nevertheless, the target volume from two-dimensional (2D) views does not provide integral information about the tumor structure. Furthermore, one image does not contain the whole information of bone structure and tumor shape. These limitations of MRI and CT have long been acknowledged, especially for PAs with complex shapes.

To overcome the limitations mentioned above, some neurosurgeons prefer the neuronavigation system, which bases on image-guided localization for assistance during the operation, particularly in the localization of tumor and internal carotid arteries [14]. However, this system has some well-known disadvantages, as it is time consuming and expensive, provoking that not all the hospitals can afford it [15]. Furthermore, the surgical planning has to be made based on two-dimensional images, which do not provide sufficient information of the surrounding structures. Therefore, the neuronavigation system still lacks sufficient spatial information to discriminate among the nidus and immediately surrounding normal vessels, such as arteries.

In light of these difficulties and the necessity to precisely define the target to avoid disastrous complications, including bleeding from the internal carotid artery or CSF rhinorrhea, we have attempted to achieve an improved interpretation of each PA’s anatomy by printing 3D prototypes of the PAs of individual patients. In this paper, we describe a technique that improves the understanding of complex macroadenomas by 3D prototyping of individual lesions.

## Materials and methods

### Patient selection

In order to investigate the value of 3D printing technology, patients with macroadenomas who underwent endoscopic endonasal surgery at the Union Hospital of Tongji Medical College, Huazhong University of Science and Technology (Wuhan, China), between January and May 2017 were selected for rereview and treatment planning. All the surgeries were completed by one experienced neurosurgeon. We selected cases in which the identification of the tumor location was not intuitive. Difficulties were due to the structure of different sphenoid sinus bones and tumor components, as, for example, the presence of too much of the sphenoid sinus septa, which made their location difficult, an unclear tumor location, and widespread tumors. Different combinations of these factors were present in different patient cases. After inspection, 20 cases that had matched tumor size, no other diseases and almost the same difficulties mentioned above were enrolled and ten cases who got the service of 3D printing technology were experimental group and ten cases who did not were control group. All patients underwent MRI and CT examinations, and age, gender, symptom, tumor type, images, postoperative complication, operation time and blood loss were recorded for each patient. The protocol for this study was reviewed and approved by the Ethics Committee of Tongji Medical College, Huazhong University of Science and Technology.

### Image acquisition and 3D printing

Imaging data from CT and MRI scans performed for clinical purposes were used for this study. To obtain the data in the stereolithography file format (*.stl) necessary for 3D printing, we used the standard DICOM image files obtained from CT and MRI. The slice thicknesses of the CT scans were 0.625 mm, and the slice thicknesses of the MRI scans were 1 mm. The freely available software MIMICS (Materialise, Leuven, Belgium) was used to convert the CT data into a 3D model. Using the program’s automated segmentation feature, the CT scans were selectively segmented using intensity thresholds to isolate the bone and the internal carotid artery. We reviewed the automatic segmentations, and an initial manual segmentation was performed to minimize the artifacts and include any of the following desired bone areas: sphenoid sinus without the anterior wall, planum sphenoid, internal carotid artery, sella turcica, upper clivus, and anterior and posterior clinoid processes. After this segmentation, we obtained the 3D images of the bone and the internal carotid artery. We performed the same procedure on the MRI data to obtain the 3D image of the tumor. Finally, we formed a virtual model by putting these two parts together according to the patient’s anatomy. The final virtual models were converted into .stl files and imported into the 3D printer. This printer used Fused Deposition Modeling Technology, which prints out the material by deposing layer by layer of acrylate resin with the layer thickness 0.245 mm.

### Model application

First, we observed the tumor site in the model and the anatomical situation in the sphenoidal sinus preoperatively. Then, the surgical approach was confirmed, and the central septum of the sphenoidal sinus was removed with the aid of an endoscopy system to expose the sella turcica and confirm the location of the internal carotid artery tumor in the sella turcica. The exposed area of the sellar floor was determined according to the contact status between the tumor and the sellar floor, and the size of the area was measured. On the day of the surgery, the tumor was excised according to the predetermined route, and the crucial anatomical sites were compared with the model. The expose area of the sellar floor was measured after tumor resection was completed.

### Statistical analysis

All the data are expressed as the mean ± SD and Mann–Whitney test or χ^2^ test was used for comparison between groups, as approprite. A p value < 0.05 was considered statistically significant. All the data analyses were performed with SPSS (version 21).

## Results

### Patients’ information

We carried out a retrospective analysis of 20 macroadenoma patients, of which 10 patients had recieved the service of 3D printing technology. In this group, 5 were males and 5 were females, with which average age was 41.20 ± 11.84 years. All patients underwent endoscopic transsphenoidal surgery, of which 9 patients underwent complete resection, while 1 patient underwent partial resection. Postoperative pathological examination revealed four cases of non-functioning adenomas, three case of PRL-secreting adenoma, three cases of growth hormone-secreting PAs. After surgery, among the 10 patients, 1 patients experienced electrolyte disorders, 1 patient experienced diabetes insipidus, and no patient experienced CSF rhinorrhea. The average operation time and blood loss were 127.0 ± 15.53 min and 143.40 ± 17.89 ml respectively.

In the group of patients who had not received the service, 5 were males and 5 were females, with which average age was 44.40 ± 8.45 years. All 10 patients were macroadenomas. All patients underwent endoscopic transsphenoidal surgery, of which 8 patients underwent complete resection, while 2 patient underwent subtotal resection. Postoperative pathological examination revealed five cases of non-functioning adenomas, three case of PRL-secreting adenoma, two cases of growth hormone-secreting PAs. After surgery, among the 10 patients, 2 patients experienced electrolyte disorders, 1 patient experienced diabetes insipidus, and 1 patient experienced CSF rhinorrhea. The average operation time and blood loss were 159.90 ± 12.31 min and 170.00 ± 29.06 ml respectively(details shown in Table 1).

**Table 1:** Summary of patients’ data

	Patients with 3D printing	Patients without 3D printing
Gender		
Male	5	5
Female	5	5
Age (years)	41.20 ± 11.84	44.40 ± 8.45
Symptom		
Asymptomatic	5	3
Headache	2	4
Acromegaly	2	1
Visual impairment	1	2
Pathological diagnosis		
Non-functioning adenoma	4	5
PRL-secreting adenoma	3	3
GH-secreting adenoma	3	2
The gross total resection	9 (90%)	8 (80%)
Postoperative complication	2 (20%)	4 (40%)
Operation time (min)*	127.0 ± 15.53	143.40 ± 17.89
Blood loss (ml)*	159.90 ± 12.31	170.00 ± 29.06

*PRL* prolactin, *GH* growth hormone,

*There is difference between them (p < 0.05)

### The 3D processing and clinical application

The PA models of the 10 patients were successfully printed, showing size, site, and surrounding structure of the tumors (detailed information shown in Tables 2, 3). The design time of the 3D images varies 2 h 10 min to 4 h 32 min. The printing time of the 3D models varies 10 h 12 min to 22 h 34 min. The appearence of model were clear and neat. The results of printing were great satisfaction. During the operation, the outline of sphenoid septation, sellar floor, carotid protuberance, tumor location was same with the models. The expose area of the model and patient of sella floor were measured as 1.01 ± 0.23 and 0.98 ± 0.31 which the difference is not statistically significant.

**Table 2:** Generalization of 3D processing

Case	Images	Design time	Material	Printing time	Satisfaction
1	CTA, MRI	2 h 43 min	Acrylate resin	11 h 36 min	Yes
2	CTA, MRI	2 h 35 min	Acrylate resin	10 h 12 min	Yes
3	CTA, MRI	4 h 32 min	Acrylate resin	22 h 34 min	Yes
4	CTA, MRI	2 h 40 min	Acrylate resin	11 h 15 min	Yes
5	CTA, MRI	2 h 10 min	Acrylate resin	10 h 20 min	Yes
6	CTA, MRI	2 h 50 min	Acrylate resin	10 h 24 min	Yes
7	CTA, MRI	2 h 56 min	Acrylate resin	13 h 37 min	Yes
8	CTA, MRI	3 h 15 min	Acrylate resin	16 h 23 min	Yes
9	CT, MRA, MRI	3 h 55 min	Acrylate resin	12 h 21 min	Yes
10	CTA, MRI	3 h 10 min	Acrylate resin	12 h 54 min	Yes

**Table 3:** Application of the 3D-printing model

	Printing model	Intraoperative observation
Sphenoid septation	Clear	Identical to the model
Sellar floor	Clear	Identical to the model
Carotid protuberance	Clear	Identical to the model
Tumor location	Clear	Identical to the model
Exposed area (cm^2^)^a^	1.01 ± 0.23	0.98 ± 0.31

^a^There is no difference between them (p > 0.05)

### Illustration cases

#### Case 1

**Fig. 1:**
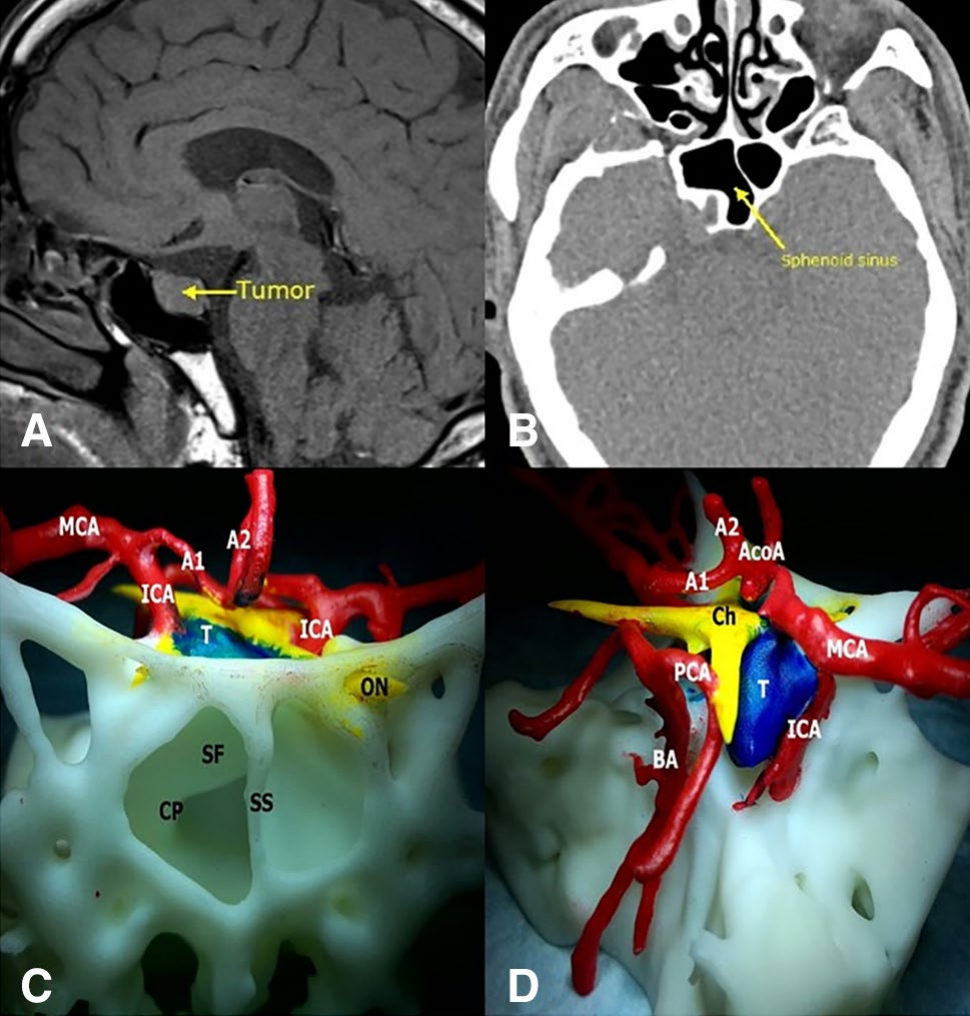
**a** MRI of the sella turcica region showing tumor lesions. The yellow arrow points to the tumor. **b** CT of the sphenoidal sinus. The yellow arrow points to the sphenoidal sinus. **c, d** Frontal and lateral views. *SF* sellar floor, *SS* sphenoid septation, *CP* carotid protuberance, *A1* A1 segment of the anterior cerebral artery, *BA* basilar artery, *PCA* posterior cerebral artery, *A2* A2 segment of the anterior cerebral artery, *Ch* chiasm, *T* tumor, *ON* optic nerve, *AcoA* anterior communicating artery, *MCA* middle cerebral artery

A 62-year-old male patient with acromegaly sought treatment at our hospital for intermittent headaches. MRI and CT showed a 1.5 × 1.0 × 1.3 cm space-occupying lesion at the sella turcica (Fig. 1A, B), which was diagnosed as a pituitary macroadenoma. Preoperative endocrine examinations showed elevated growth hormone levels, and the patient was advised to undergo endoscopic transsphenoidal surgery. We employed 3D-printing technology to reconstruct a model of the patient’s tumor (Fig. 1C, D). This model has been used as demonstration object to actively communicate the patient’s condition with his relatives, which led to good communication results. On the model, we also planned the surgical approach and practiced the surgical manipulation, which provided important guidance for the surgery, leading to full tumor resection. Pathological examination showed that the patient’s tumor was a growth hormone-secreting PA. No complications occurred after the surgery, and the patient was successfully discharged.

#### Case 2

**Fig. 2:**
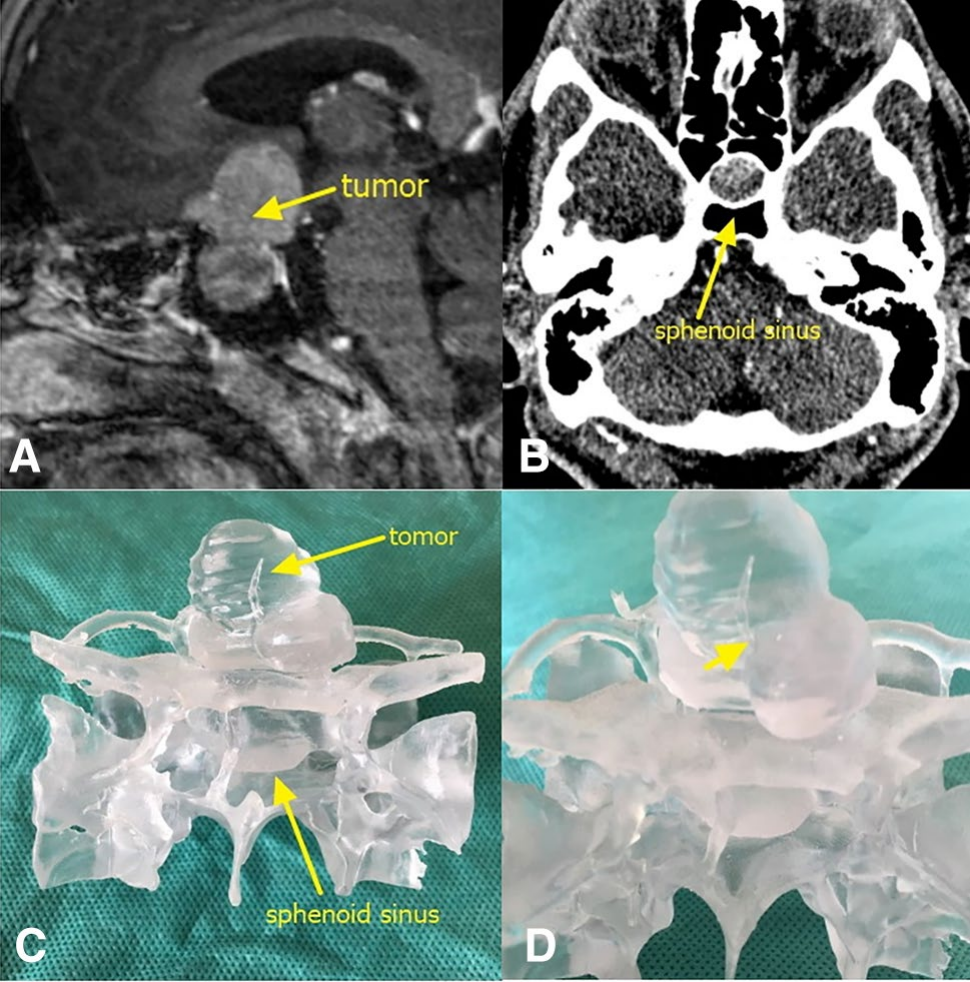
**a** MRI of the sella turcica region showing tumor lesions. The yellow arrow points to the tumor. **b** CT of the sphenoidal sinus. The yellow arrow points to the sphenoidal sinus. **c** Frontal view of the 3D-printed tumor model. **d** Enlarged image of a part of the tumor. The yellow arrow shows that the tumor wraps around the left side of the anterior communicating artery

A 28-year-old female patient with acromegaly sought treatment at our hospital for poor glucose control. MRI and CT showed a 3.9 × 2.4 × 3.3 cm space-occupying lesion in the sella turcica region (Fig. 2A, B). The patient was diagnosed with pituitary macroadenoma, and preoperative endocrine examinations showed elevated growth hormone levels. We employed 3D-printing technology to reconstruct a model of the patient’s tumor (Fig. 2C, D) and used the model as a demonstration object to actively communicate the patient’s condition with his relatives. We recommended to the patient to undergo first a transsphenoidal surgery to remove the intrasellar tumor and then a craniotomy to remove the suprasellar tumor. We obtained good condition communication results. At the same time, we planned the surgical approach on the model and practiced the surgical manipulation, which provided important guidance for the surgery. Pathological examination showed that the patient’s tumor was a growth hormone-secreting PA. No complications occurred after the surgery, and the patient was successfully discharged. Three months later, the patient went for craniotomy at our hospital, and the tumor was fully resected.

## Discussion

3D-printing technology is an emerging technology with wide potential applications in various occupations and industries. As it is highly precise and allows personalization, 3D-printing technology has started to be applied in medicine in recent years [16, 17]. In neurosurgery, 3D-printing technology can provide models for the patients’ disease characteristics, such as skull defects [18], brain tumors [19], intracranial aneurysms [20], and intracranial vascular malformations [21]. 3D-printing technology provides tools that aid in the resection of tumors, such as positioning models for the resection of tumors in brain functional regions [22] and navigation models for spinal surgery [23, 24]. 3D-printing technology provides us with opportunities for surgery practice on the model, such as simulation of cerebral aneurysm clipping surgery [25] and modelling of intraventricular tumor resection [26]. Benign pituitary tumors are located in the midline skull base region and present one of the most commonly seen tumors in the brain. These tumors are deep and anatomically complex. Currently, the main surgical treatment used for tumor resection is transsphenoidal surgery. Although the surgical approach is carried out via natural channels (bilateral nasal cavity), decreases injury to the patient, and is characterized by less damage, less complications, and faster recovery, clinicians are usually confused by the complex anatomical structures, which limit the promotion of this technique. In fact, in some cases, a digital 3D reconstruction of the key anatomy is sufficient for the experienced surgeon to study or it is not necessary to have a tangible 3D printed model, but in some complicated cases, 3D printed model provided us a valuable tools for surgical or anatomic considerations. In this artical, we combined endoscopic surgery with 3D-printing technology and obtained good clinical outcomes, which are reported in the following.

### Model selection

Previously, many papers about the use of 3D printing in medicine mentioned that 3D-printed models are time and money consuming [27, 28], which impedes the application and promotion of 3D printing in medicine. In order to solve this problem, we limited the printed model to reflect the surrounding anatomical structures of the tumor. This facilitates image reconstruction in the computer and can reduce the loss of local details. In addition, it facilitates rapid printing, reduces material wastage, and reduces economic costs. In transsphenoidal surgery, the key to the success of the entire surgical process is the local anatomical status in the sphenoidal sinus and the relationship of the tumor with surrounding blood vessels and nerves. Our printed model nicely visualized these structures, which fulfilled our requirements. The color of the entire printed model was white. In order to better visualize the tumor, blood vessels, and bones, we used different labeling colors to facilitate the communication with the patients’ relatives on the patients’ conditions. The model printing time ranged from 10 h 12 min to 22 h 34 min, which was completely within the time frame that we can control. Therefore, choosing the local area of the model for printing has some advantages.

### Surgical planning and clinical results

**Fig. 3:**
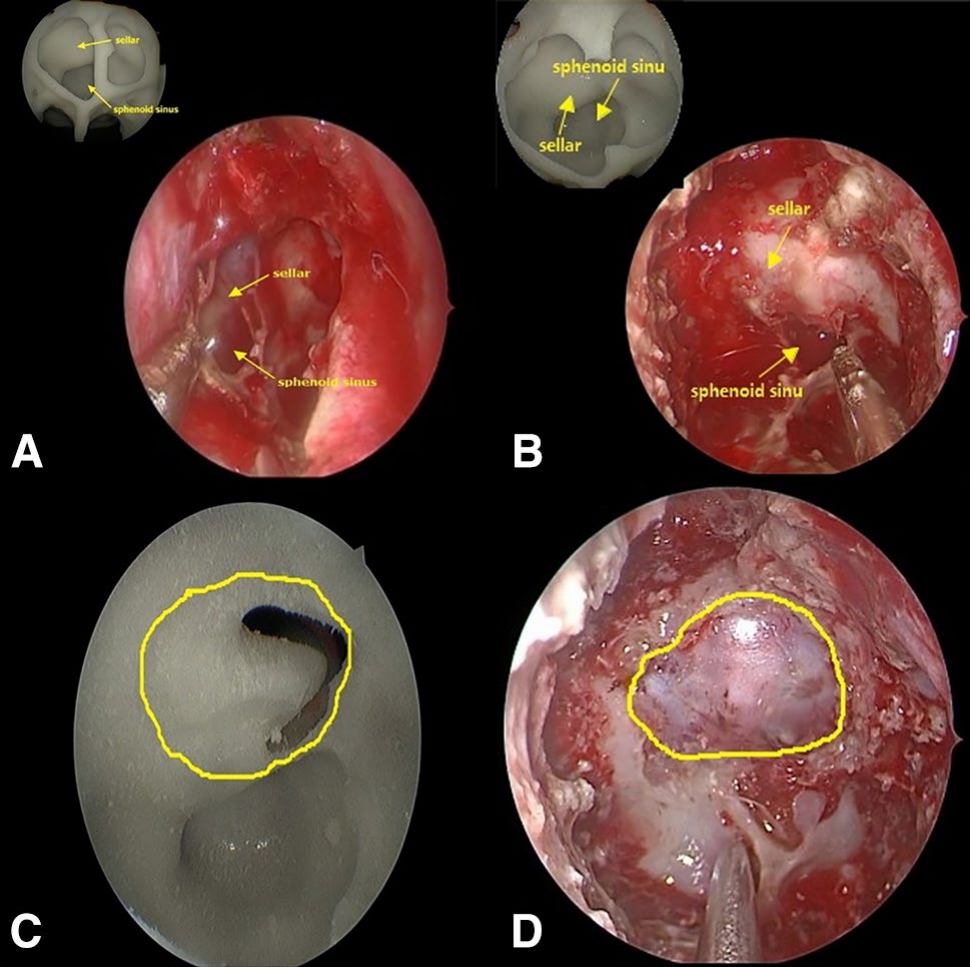
**a** Endoscopy of the anatomical status of the sphenoidal sinus and copmparing with the model. **b** Exposed sellar floor after removal of the septal bone and copmparing with the model. **c** Tumor site after removal of the sellar floor bone. **d** Exposed area of the sellar floor observed in surgery

**Fig. 4:**
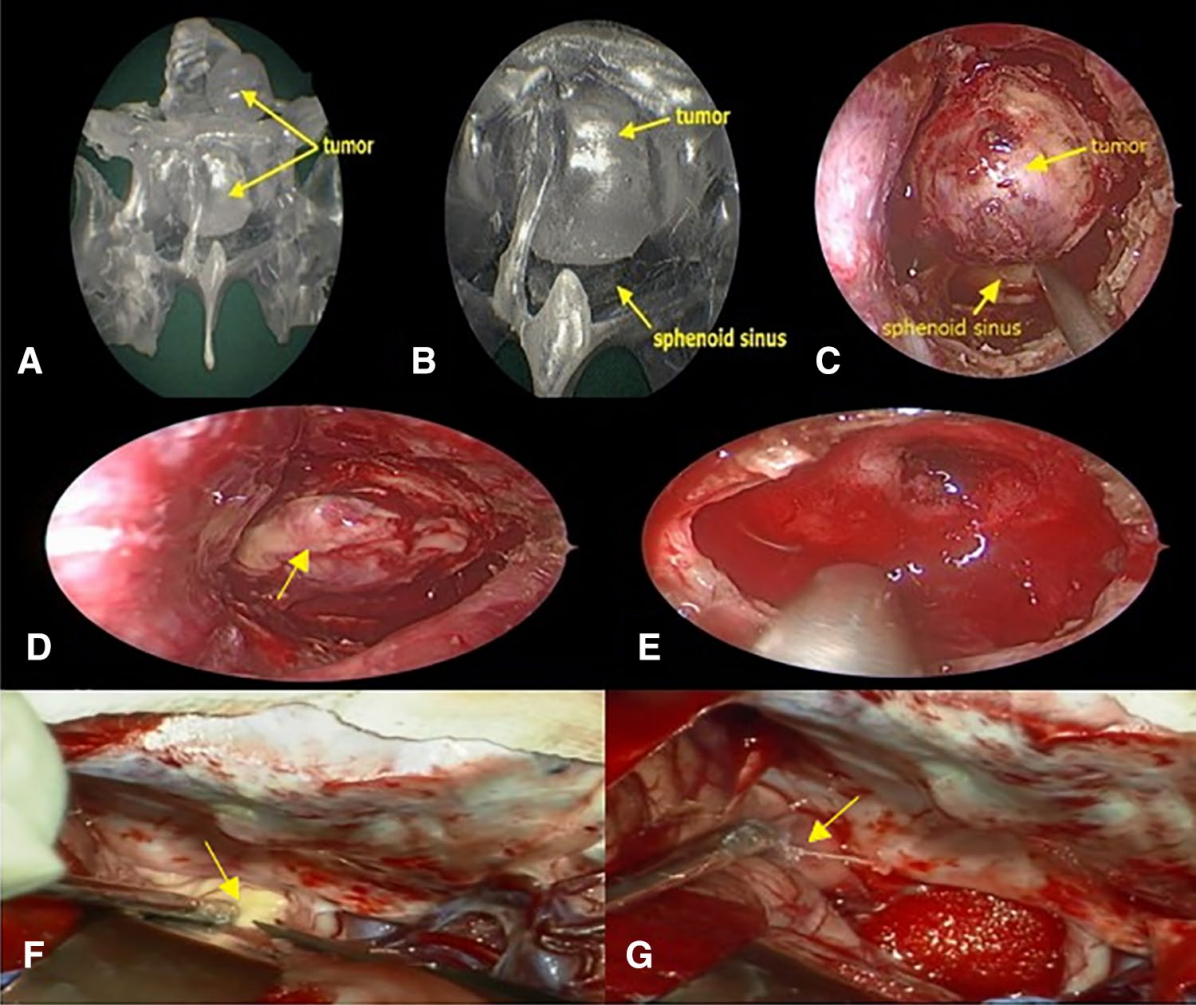
**a** Endoscopy of the tumor model. **b** Close-up of the anatomical status of the sphenoidal sinus under endoscopy. **c** Anatomical status of the sphenoidal sinus observed in surgery. **d, e** The tumor under the sellae diaphragma was exposed and had been totally removed. **f, g** The tumor above the sellae diaphragma had been partially removed by craniotomy (yellow arrow referred to the tumor)

The models we printed reflected the situation of the sphenoidal sinus bone, the tumor, and the blood vessels in the sella turcica region. This realistically exhibited the anatomical relationships at the surgical site and provided us with a foundation for obtaining good surgery outcomes. Through the model, we understood the orientation of the intraseptal bone in the sphenoidal sinus, the morphology of the sellar floor, and the route of the internal carotid artery in the sellar floor, and roughly determined the site of the tumor before surgery. Subsequently, we performed simulated endoscopic manipulations on the model to remove the septal bone in the sphenoidal sinus, fully expose the sellar floor, and show the tumor site. In addition, we also measured the size of the removed sellar floor area to improve the precision of our surgery. In case 1, it was difficult to outline the 3D image of the tumor from 2D images. Therefore, 3D-printing technology was used to realistically restore the relationships of the spatial location of the tumor. Before surgery, we planned the exposure of the sellar floor to reduce damage to adjacent important structures (Fig. 3), which reliably assured good surgical outcomes. In case 2, the tumor exhibited an extremely irregular morphology, and the suprasellar tumor growth region was relatively extensive, as it invaded the anterior skull base and wrapped around the left side of the anterior communicating artery (Fig. 4). Therefore, dissecting the tumor was relatively complex. After careful consideration, we decided to adopt a two-stage surgery. Firstly, endoscopic transsphenoidal surgery was used to remove the intrasellar tumor, while the sella turcica septum was not resected to prevent CSF rhinorrhea. After the patient had recovered well from the surgery, a craniotomy was conducted to excise the suprasellar tumor. Results proved that our strategy was successfull. This shows that 3D-printed models are not only useful for pre-surgery practice, but can also visualize the tumor in the sella turcica region. Therefore, 3D-printed technology provides valuable assistance in making surgery decisions.

In order to further study the clinical value of 3D printing, we analyzed the data of patients with the same size of the pituitary adenoma, and made statistical analysis from the time of operation and the amount of blood loss during the operation. From the Table 1, we can see that the patients with the help of 3D printing technology had a less operation time (p = 0.007) and blood loss (p = 0.009). As a result, the experimental group had a less postoperative complications rate (20% vs. 40%). Although the datas need more support, but the 3D model really make surgeon become more confidence and aware of the vital anatomy structures.

### Patient communication and education

It is widely known that neurosurgeon is a challenging career with high risks and intensity. Surgery risks are higher due to the importance of the lesion site and the complexity of the intracranial structures. However, during communication of the patient’s condition with relatives with limited medical knowledge it is difficult to reach a consensus, as physicians often use professional terminology to accurately explain the patient’s condition. To improve the relatives’ understanding of the patient’s condition, objects are used to support the professional description of the medical contents. However, this often results in relatives not fully comprehending the patient’s condition. On the other hand, by demonstrating color-labeled models to the relatives and informing them about the surgery process and possible postoperative complications, the relatives fully understood our surgical approach and intention and took careful decisions. This shows that our models can produce good condition communication results. For a neurosurgery student or doctor with little clinical practice experience and shallow understanding of anatomical structures at crucial sites, an improvement of their surgical techniques is frequently limited to learning from precepts and examples from senior physicians during surgery. They also often have a shallow understanding of the choice of surgery options for different methods. By showing the 3D-printed models of different tumor patients to such amateurs, a more accurate and deeper impression of key anatomical locations and surrounding blood vessels and nerves can be achieved, which will improve their medical skills as well as their understanding for making better decisions about surgery methods.

### Skull base construction

CSF rhinorrhea has always been one of the major complications in transsphenoidal surgery. It can be caused by inappropriate surgical manipulations and excessively large skull base defects. In order to prevent the occurrence of this complication, we delineated the exposed area of the sellar floor when the sellar floor was fully exposed. This method has two advantages: firstly, it not only reduces damage to surrounding tissue but also avoids CSF rhinorrhea due to excessively large skull defects; secondly, it provides us with supporting data. Once CSF rhinorrhea occurs, we can select materials with a suitable area for repair according to the size of the exposed area.

## Conclusions

This study presents the detailed report of the application of 3D-printing technology in transsphenoidal surgery for macroadenomas. All patients had good postoperative recoveries, which proves that the application of 3D-printing technology in transsphenoidal surgery has good prospects. The 3D-printed models allowed understanding the anatomical structures of pituitary tumors at the sella turcica region before the surgery, which was particularly advantageous in the pathology of more complex anatomical structures. Furthermore, this model provided realistic disease characteristics for surgery planning and preoperative practice as well as a foundation to obtain good surgery outcomes. We believe that with its continuous development, 3D-printing technology will be applied in clinical practice in the near future.

## Abbreviations

MRI: Magnetic resonance imaging
MRA: Magnetic resonance angiography
CT: Computed tomography
CTA: CT-angiography
DICOM: Digital imaging and communications in medicine
